# Evolving from Reactive to Proactive Medicine: Community Lead (Pb) and Clinical Disparities in Pre- and Post-Katrina New Orleans

**DOI:** 10.3390/ijerph110707482

**Published:** 2014-07-21

**Authors:** Howard W. Mielke, Christopher Gonzales, Eric Powell, Paul W. Mielke

**Affiliations:** 1 Department of Pharmacology, Tulane University School of Medicine, New Orleans, LA 70112, USA; 2 Lead Lab, Inc., New Orleans, LA 70119, USA; E-Mails: powellet2@gmail.com (C.G.); chrisgc99@gmail.com (E.P.); 3 Department of Statistics, Colorado State University, Fort Collins, CO 80523, USA; E-Mail: rrmielke@aol.com

**Keywords:** soil lead mapping, Pb loading, clinical Pb testing, exposure intervention

## Abstract

In 2012 the U.S. Centers for Disease Control (CDC) set the blood Pb reference value at ≥5 µg/dL. Clinical analysis of children’s blood Pb levels is the common way to diagnose environmental Pb contamination, and intervention ensues with education and household dust cleanup. Recent review indicates that education and household dust cleanup are not effective at reducing children’s Pb exposure. Here we review mapping environmental Pb and children’s blood Pb response as an alternative approach for proactive Pb dust intervention. New Orleans was divided into a high (≥100 mg/kg) and low (<100 mg/kg) soil Pb communities. The children’s blood Pb prevalence ≥5 µg/dL for the high and low Pb domains were 58.5% and 24.8% respectively pre-Katrina *vs.* 29.6% and 7.5% post-Katrina. Elevated soil Pb (mg/kg) and consequently Pb loading (µg/square area) permeates the high Pb domain and outdoor locations lack Pb dust safe play areas. The U.S. EPA 400 mg/kg soil Pb standard poses an outside Pb dust loading burden >37 times larger than allowed on interior residential floor environments. Environmental Pb dust is decreasing because of the transfer of large quantities of low Pb soil into selected communities. City-scale soil Pb mapping is an alternative diagnostic tool that provides information for planning proactive medicine to prevent clinical Pb exposure in the first place.

## 1. Introduction

“*Sometime in the near future it probably will be shown that the older urban areas of the United States have been rendered more or less uninhabitable by the millions of tons of poisonous industrial lead residues that have accumulated in cities during the past century*.”

*—Clair Cameron Patterson* [1]

The common clinical approach to lead (Pb) exposure is reactive because it relies on results of children’s blood Pb level (BPb) as a trigger for intervention actions. The reactive approach assumes the existence of a safe BPb level below which there is no medical harm to children. Diagnosis depends on available instruments to analyze BPb. Each BPb reduction was preceded by improvements of available analytical instruments for diagnosing BPb [2]. From the 1960s to the present the guidelines (given in micrograms per deciliter or µg/dL) underwent remarkable reductions [3]. During the 1960s the guideline was 60 µg/dL, from 1970–1985 it was 30, in 1985–1991 it was reduced to 25, in 1991–2012 the guideline was reduced to 10 µg/dL. With each analytical improvement the clinical research on lead became more refined to the degree that it became evident there was no known safe level of Pb exposure [2]. In 2012 CDC made a fundamental change by replacing the word “guideline” with “reference value” [4]; the current BPb reference value is 5 µg/dL. The National Health and Nutrition Examination Survey (NHANES) results determine the reference value; the empirical reference value is defined as the BPb level of 97.5 percentile of the children [4]. The change by CDC emphasizes the need for primary prevention.

Intervention is based on the intensities of exposure according to children’s BPb [5]. The main method of intervention focuses on education and cleanup of household Pb dust. It is generally assumed that the cause of lead dust is Pb-based paint. All medical interventions require continuous review to assure that evidence-based medicine is effective in achieving the intended clinical goals. The Cochrane Collaboration recently reviewed the literature on education and household interventions for preventing lead exposure [6]. The review evaluated 14 education and dust control studies, and based on meta-analysis noted that education and household interventions are not effective at reducing BPb levels in young children [6]. If the standard intervention approach is not effective then what alternative methods are available to intervene and reduce BPb exposure in young children?

This overview of New Orleans research examines the sources of lead accumulation in the environment, the urban pattern of soil Pb and Pb loading together with childhood Pb exposure, and puts forward a proactive medical approach for diagnosing conditions of a preventable, chronic childhood disease that afflicts society. The New Orleans research was an extension of empirical studies that began in the 1970s in Baltimore, Maryland, continued in the 1980s in Minneapolis and Saint Paul including various smaller cities of Minnesota, and progressed in Louisiana in the 1990s [7,8,9,10].

## 2. Processes Section

### 2.1. Lead-based Paint Coatings

Lead-based paint coatings are one of the legacy sources of Pb that contaminated the environment. Old homes in New Orleans are often coated with Pb-based paints. Because Pb-based paints were priced according to their Pb content, wealthier communities have larger Pb contents in paint than poorer communities where cheaper, low or non-Pb paints were used [11,12]. Empirical accounting of the Pb dust quantities attributable to paint on homes is not well documented. New Orleans studies of metal content of intact paint revealed a wide range of Pb and Hg content in exterior as well as interior paints of residential housing stock [11,12].

### 2.2. Lead-additives to Vehicle Fuels

Accounting for the Pb additives dispersed from fuels as dust is not an empirically common practice. When accounting for Pb-additives in vehicle fuels is done then new perspective about this major source of Pb dust is obtained for urban environments. Along with accounting for Pb-based paint dust, the New Orleans studies account for Pb additives in fuel as part of the overview of Pb dust contamination of the urban environment.

### 2.3. Soil Lead Map

Soil lead (SPb) was surveyed and mapped throughout the entire metropolitan area. Mapping the combination of SBb and BPb by communities of metropolitan New Orleans is one contribution these studies have made to the scientific literature [13,14,15,16].

### 2.4. Blood Lead Results

Blood lead results were collected by the Louisiana Childhood Lead Poisoning Prevent Program. The BPb data was stratified by census tracts and this provided the means for evaluating lead exposure within the context of New Orleans before and after major flooding of 80% of the city by Hurricane Katrina. The combination of SPb and BPb provides information about Pb exposure in context of various New Orleans communities [16].

## 3. Results and Discussion

**Figure 1:**
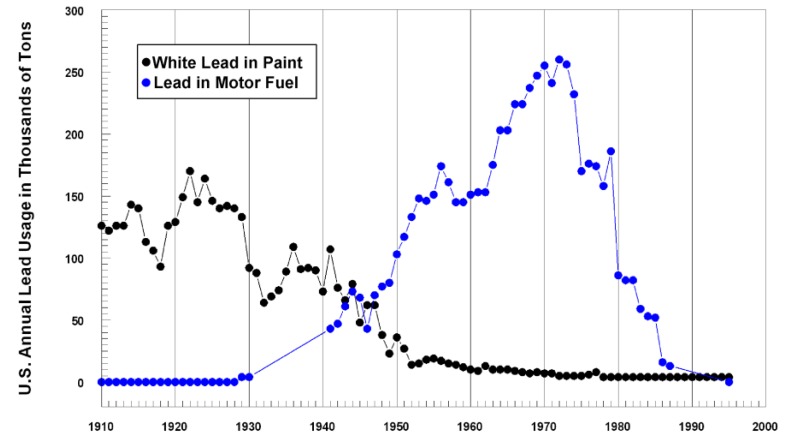
Annual U.S. tonnages of Pb used for the manufacture of paint and vehicle fuel (gasoline) additives. The major commercial use of Pb is for the manufacturing of batteries, but this use does not generally produce large quantities of Pb dust in residential areas. The use of Pb in paint and vehicle fuel resulted in widespread Pb dust contamination of residential communities. Note that Pb-based paint use peaked in the 1920’s, and was restricted to 600 mg/kg for interior use in 1978. Use of Pb additives in highway fuel peaked in the 1960’s and 1970’s, and was restricted beginning in the mid-1970’s, further restricted in 1986, and finally banned in 1995 for highway vehicle use. Pb is still added to fuel (Avgas) for small aircraft. *Source*: Updated from a previous graph [14].

To comprehend the urban dimensions of the legacy of Pb it is essential to account for the amount and pattern of dispersal of Pb containing products that contaminated U.S. environments. Approximately equal masses of Pb were produced for Pb-based paint and Pb additives in vehicle fuels. The tonnages used in both products annually were graphed by Mielke and Reagan [14,17] and updated in Figure 1 [18,19]. This depiction of commercial quantities of Pb used in U.S. paint and fuel is similar to a graph published independently by Clark *et al.* [20].

### 3.1. Lead-based Paint Coatings

Exterior Pb-based paints generally contained larger amounts of Pb than interior paint coatings [12]. To estimate the amount of Pb on residences we scraped off and weighed the exterior paint on one residence, and then we collected paint samples from many residences. Assuming that all of the paint was sanded off of all 86,000 houses we liberally estimate that 1,000 metric tons of Pb dust would contaminate the New Orleans environment [21,22].

### 3.2. Lead Additives in Gasoline

The quantities of Pb used as additives in vehicle fuel were calculated by linking Pb in fuel per year, the amounts of Pb in different grades of petrol, and the portion of travel within New Orleans compared with total travel in the Louisiana, and the proportion of Pb trapped in engine oil, engine components and the exhaust system were subtracted to arrive at the quantity of Pb exhausted into the atmosphere [23]. Calculating the quantity of Pb exhausted by vehicles in New Orleans resulted in at least 10,000 metric tons of lead [21,22]. The quantities of Pb used in fuel are shown for the period from 1928 until the complete ban in 1995. Although Pb is no longer used as an octane enhancer in highway use vehicles, Pb additives continue to be used in aviation fuels (Avgas) for piston engine aircraft.

### 3.3. Map of Soil Pb (µg/g) in New Orleans

**Figure 2:**
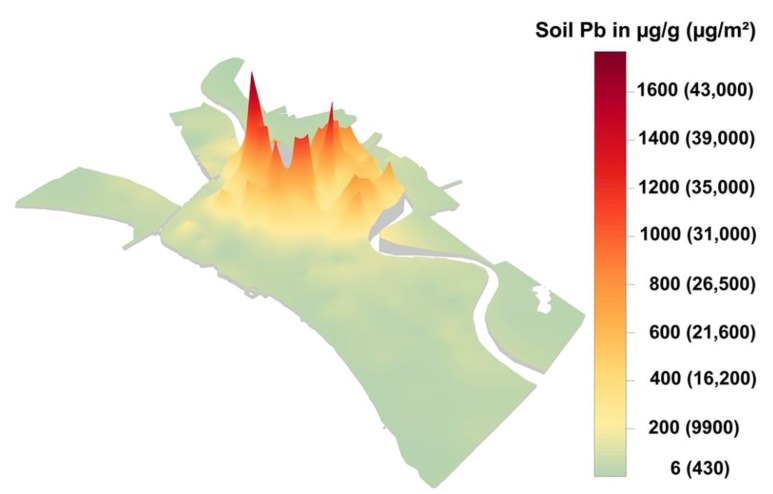
The three dimensional map of New Orleans soil showing two methods for measuring Pb. The numbers in the legend without parentheses are the soil Pb contents given in units of µg/g. The numbers in the legend with parentheses given in units of represent the Pb loading of the soil surface as measured with the PLOPS sampler. The Pb standard for soil is 400 µg/g which is equivalent to soil Pb loading of 16,200 µg/m^2^ and over 37 times more Pb than the 431 µg/m^2^ U.S. EPA Pb loading standard for interior floors.

The lead map was created by systematically by collecting (*n* = 5,467) surface soil samples (2.5 cm deep), stratified by census tracts (*n* = 286) within residential communities of the city as described in a previous article in this journal [15]. Figure 2, shows a three dimensional view of both Pb content (in µg/g) and Pb loading (µg/m^2^) of soil.

### 3.4. Map of Pb Loading (µg/m^2^) in New Orleans

Lead loading refers to the amount of Pb on the soil surface. Interior environments have the benefit of hard surfaces making it relatively easy to quantify Pb loading by measuring an area and wiping it [24]. Outdoor soil is three-dimensional and children experience Pb by placing their hands on the soil surface. Lead loading of outdoor soil is not normally quantified. We undertook measuring Pb loading on soil surfaces by developing a simple tool, the Potential Lead on Play Surfaces (PLOPS) sampler [25]. Basically the PLOPS sampler is a soft plastic storage bag containing 1 kg of water with a wipe attached on the bottom; the wipe is the same type of wipe used for measuring surface loading on floors in indoor environments. The PLOPS with the wipe attached on the bottom is used by positioning it on the soil surface and twisting it a quarter turn. Then the wipe is detached, placed into a sample cup, and taken to the laboratory for extraction and analysis. The Pb loading of the soil surface is calculated from the surface area of the wipe on the bottom of the PLOPS, and the Pb content of wipe is used to arrive at the µg/m^2^ of Pb loading [25]. The PLOPS was used to sample a soil site first and then a soil sample was collected from the same site. We sampled sites in high Pb and low Pb communities for both Pb loading and soil Pb content throughout the city. Thus surface loading results from the PLOPS samples and the Pb content of soil samples were obtained for the same locations. Based on the analytical result of the soil Pb content and the Pb loading (*n* = 136) the relationship of the two sets of analytical results follows [25]:

(x = 43.74 + 24.85y^0.69^) (1)

Agreement = 0.62, *p*-value << 0.0000001 (2)

where x = the quantity of the Pb loading of the soil, and y = Pb content of the top 2.5 cm of the soil.

### 3.5. Soil Pb Content and Soil Pb Loading Map of New Orleans

Figure 2 is particularly important because it shows the soil Pb content along with the corresponding soil Pb loading of the communities of New Orleans. The map illustrates the difference between common measurements of soil Pb and the quantities of Pb on the soil surface that children are likely to encounter during outdoor play activities. The 2013 standard for dust Pb loading on interior floors is ~431 µg/m^2^ (40 µg/ft^2^ based on U.S. standards). Note that in Figure 2 soil containing the minimum Pb of 6 µg/g has a Pb loading value of ~430 µg/m^2^ and that soil containing 400 µg/g, the U.S. HUD, EPA and CDC standard, has a Pb loading value of ~16,200 µg/m^2^. This means that the U.S. EPA soil Pb standard poses a Pb loading value in outside environments which is over 37 times larger than the Pb dust standard allowed on floors within home interior environments.

Soil Pb and lead dust are invisible to the senses. The map visualizes the extent of Pb dust dispersion in New Orleans. The relationship between SPb and BPb has also been evaluated in New Orleans and the main observation is that in communities where SPb is <100 µg/g children respond with rapid increase in BPb while in communities where SPb is ≥100 µg/g children respond with a lower rate of increase than less contaminated communities [16,26]. Also Pb loading places the issue into context of the exposure potential of communities and illustrates why contaminated soils pose such large difficulties to the urban population, especially children living in the highly contaminated soils of the interior of the city [13,16,25]. For example, in pre-Katrina New Orleans the prevalence of children’s blood Pb ≥ 5 µg/dL for the areas of the city contaminated with ≥100 µgPb/g was 58.5% compared with a prevalence of 24.8% in the parts of the city contaminated with <100 µgPb/g [16]. After Hurricane Katrina there was an important reduction of children’s blood Pb in New Orleans. In the areas of the city containing ≥100 µgPb/g the prevalence of ≥5 µg/dL declined to 29.6% whereas in the areas of the city with <100 µgPb/g the prevalence of BPb ≥ 5 µg/dL declined to 7.5% [16]. Children living in all areas of the city remain at risk to Pb exposure, but the exposure is particularly intense in the interior of the city. Note that the decline in BPb after Katrina was by a factor of 2 in areas ≥100 µgPb/g and a factor of almost 3 in the areas of the city with <100 µgPb/g soil. There is no margin of safety for children in any community of the city. The critical societal issues connected with exposure of infants and toddlers to Pb are chronic health outcomes ranging from learning and behavioral problems for children to Alzheimer’s disease (AD) and violence later in the lifespan [22,27,28,29].

## 4. Conclusions

When Clair C. Patterson declared “Sometime in the near future it probably will be shown that the older urban areas of the United States have been rendered more or less uninhabitable by the millions of tons of poisonous industrial lead residues that have accumulated in cities during the past century…” [1] his statement met with skepticism. Recent research has demonstrated the underlying reality of Pb exposure, and the results challenge society to take corrective actions concerning Pb exposure especially in urban environments where the majority of humanity is housed. Basically the capacity to mass produce and disperse exotic chemicals outstrips the ability to clinically diagnose their health impacts. Clinicians use a reactive approach which first tests children’s blood Pb and then intervenes to find and treat the results of environmental Pb exposure. However because of their DNA programing, fetuses, infants and toddlers are extraordinarily sensitive to environmental Pb contamination. Environmental Pb acts to epigenetically reprogram DNA with profound clinical and chronic health consequences. One way out of this conundrum is to focus on a proactive approach which detects highly contaminated communities in the first place and then intervenes to diminish exposure sources and create healthier communities. An example of a diagnostic map is illustrated for New Orleans in Figure 2. In addition to maps as a tool for diagnoses of community Pb contamination, actions are needed to develop appropriate and cost effective measures that implement large area soil Pb intervention projects. Landscaping projects which transfer substantial amounts of low Pb soil into Pb contaminated communities have been undertaken [30,31]. The purpose of the intervention is to reduce Pb loading and the burden of Pb dust exposure and their health consequences to people who live in contaminated communities of the city.
